# The density of anthropogenic features explains seasonal and behaviour-based functional responses in selection of linear features by a social predator

**DOI:** 10.1038/s41598-020-68151-7

**Published:** 2020-07-10

**Authors:** Karine E. Pigeon, D. MacNearney, M. Hebblewhite, M. Musiani, L. Neufeld, J. Cranston, G. Stenhouse, F. Schmiegelow, L. Finnegan

**Affiliations:** 1 fRI Research, 1176 Switzer Drive, Hinton, AB Canada; 2 Department of Ecosystem and Conservation Science, W.A. Franke College of Forestry and Conservation, University of Montana, Missoula, MT USA; 3 Department of Biological Sciences, Faculty of Science, University of Calgary, Calgary, AB Canada; 4 Parks Canada, Jasper National Park, Jasper, AB Canada; 5 Arctos Ecological Consultants, Edmonton, AB Canada; 6 Department of Renewable Resources, University of Alberta, Edmonton, AB Canada; 7 Yukon Research Centre, Yukon University, Whitehorse, Yukon, Canada; 8 Present Address: Geomatics and Landscape Ecology Laboratory (GLEL), Carleton University, Ottawa, Canada; 9 Present Address: Wildlife Research Division, Environment and Climate Change Canada, National Wildlife Research Centre, Ottawa, ON Canada

**Keywords:** Behavioural ecology, Boreal ecology, Conservation biology

## Abstract

Anthropogenic linear features facilitate access and travel efficiency for predators, and can influence predator distribution and encounter rates with prey. We used GPS collar data from eight wolf packs and characteristics of seismic lines to investigate whether ease-of-travel or access to areas presumed to be preferred by prey best explained seasonal selection patterns of wolves near seismic lines, and whether the density of anthropogenic features led to functional responses in habitat selection. At a broad scale, wolves showed evidence of habitat-driven functional responses by exhibiting greater selection for areas near low-vegetation height seismic lines in areas with low densities of anthropogenic features. We highlight the importance of considering landscape heterogeneity and habitat characteristics, and the functional response in habitat selection when investigating seasonal behaviour-based selection patterns. Our results support behaviour in line with search for primary prey during summer and fall, and ease-of-travel during spring, while patterns of selection during winter aligned best with ease-of-travel for the less-industrialized foothills landscape, and with search for primary prey in the more-industrialized boreal landscape. These results highlight that time-sensitive restoration actions on anthropogenic features can affect the probability of overlap between predators and threatened prey within different landscapes.

## Introduction

The industrial footprint is widespread in forest ecosystems worldwide, and has had significant impacts on forest structure, heterogeneity, and fragmentation^1^. With a changing mosaic of available forage and prey species^2^, resting habitats^3^, accessibility and availability of travel routes^4^, and with increased year-round human activity within forested ecosystems^5^, industrial activity has altered the composition and species interactions of wildlife inhabiting forest ecosystems^6^. Although human-induced changes on forest ecosystems can enhance availability and abundance of forage for wildlife that thrive in early successional forests^2^, these changes can also have a multitude of negative impacts on wildlife species such as reduced nesting opportunities and cover for species that rely on mature and contiguous forests^7^.

Anthropogenic linear features can fragment forested habitats, reduce the amount of effective habitat for wildlife species, and increase direct mortality of wildlife^8^. Linear features are also associated with facilitated access, movement, and travel efficiency for predators^9,10^, and with increased predation risk for ungulate prey species^11^. Legacy seismic lines, hereafter “seismic lines”, are linear features of particular significance within the boreal forest of Canada. Seismic lines are 5–15 m wide and were built for oil and gas exploration starting in the early 1950s. They are pervasive throughout the boreal forest of western Canada^12,13^, and have been slow to regenerate because of ground compaction and altered hydrology from mechanical damage during construction, and from continued compaction and physical damage to soil and vegetation from ongoing motorized traffic^14,15^. Restoration of seismic lines is a key component of recovery planning for threatened species such as woodland caribou^16–18^, and there is a growing need to understand how attributes of seismic lines (i.e., the height and species composition of vegetative re-growth, soil attributes, and physical characteristics) influence how wildlife use these seismic lines. Understanding how attributes of seismic lines influence their use by wildlife can be used to effectively prioritize restoration in areas where it is most needed^9,10,19,20^, and to evaluate the effectiveness of potential restoration actions^16^.

Although specific types of anthropogenic features (i.e., roads or seismic lines) can have profound impacts on animal use of landscapes^8^, the effects of several types of anthropogenic features from multiple industrial activities (i.e., roads, well sites, and harvest blocks combined) have been increasingly scrutinized, e.g.^12,21,22^. Industrial activities can alter the spatial composition and arrangement of diverse landscape features (i.e. landscape heterogeneity), have compounding effects on the way that wildlife perceive disturbance features, can occur at multiple scales, and can influence the functional response in habitat selection^23,24^. Typical habitat selection models make the implicit assumption that selection stays constant as availability changes. Instead, an increasing number of studies demonstrate a functional response in habitat selection, where selection is influenced by changes in the availability of specific habitat types^25^. For example, Houle et al*.*^24^ looked at seasonal wolf response to harvest blocks and roads, and found that wolf selection for regenerating harvest blocks decreased with increasing densities of harvest blocks, and with increasing local road densities. Combined, landscape heterogeneity and cumulative effects of industrial activity can therefore add levels of complexity to our understanding of how wildlife perceive their environment by altering functional responses in habitat selection, which in turn can influence predator–prey dynamics in complex landscapes.

Predation is a major regulator of prey populations^26^, and the ability to find (encounter) and kill (attack) prey is influenced by prey distribution and predator density, but also by attributes of the landscape such as topography, and the density and layout of anthropogenic features^27^. As apex predators, wolves have the ability to limit herbivore populations, and previous research has shown that wolves select linear features such as seismic lines as travel corridors to improve access to prey, and move faster on, or near, seismic lines, particularly during snow free months^10,19^. Wolf selection for seismic lines is therefore believed to increase predation risk for threatened species such as caribou. Attributes of seismic lines such as vegetation height^10,19^, and surrounding habitat configurations including landcover types and densities of anthropogenic features can affect how wolves use seismic lines. Understanding how wolves respond to these heterogeneous landscapes could therefore be crucial in developing effective restoration actions aimed at limiting wolf use of seismic lines, and ultimately, predator-caused mortalities.

Our objectives were to understand whether attributes of seismic lines, including vegetation height and soil wetness, influenced the selection patterns of wolves, and whether the density of anthropogenic features surrounding seismic lines led to scale-specific functional responses in habitat selection within two contrasting landscapes (i.e., two heterogeneous landscapes). We predicted that wolves would prefer (1a) areas near seismic lines, (1b) areas near lower vegetation height seismic lines compared to higher vegetation height seismic lines because low vegetation facilitates travel^9,10^, and (1c) low vegetation seismic lines more during snow-free seasons when prey are diffuse and increased use of seismic lines for travel is likely to be beneficial^10,28^. Previous research has also shown that when at high elevation, wolves increasingly select linear features, presumably because travel efficiency is greater on linear features in rugged terrain associated with high elevation^11^. Following these findings, we predicted that wolf selection for low vegetation height seismic lines would increase with (2a) elevation, and (2b) more rugged terrain. Also, primary prey such as moose, deer, and elk forage in open-wet forested areas with abundant early seral forage^29^ while caribou may use wetlands to try and avoid predators^30^. Although travel efficiency should be lessened in wet areas because of increased sinking depth, wolves have been shown to consistently select wet meadows for rendezvous sites, presumably because of abundant hiding cover, water, and food supply for pups with limited mobility^31^. Therefore, we predicted that year-round, but especially during the rendezvous season, wolves would prefer (3a) areas near wetter seismic lines more than areas near drier seismic lines to facilitate successful pup rearing, but that selection for areas near wet seismic lines would be (3b) greater where seismic lines had low vegetation because wet areas with low vegetation are associated with high-quality browse species for ungulates targeted by wolves^32,33^, or (3c) occurred in open young forests because wet early seral forests are also indicative of abundant high-quality browse species for primary prey^34–36^. Finally, because changes in availability can lead to functional responses in habitat selection^25^, we also predicted that (4) wolf selection for areas near low vegetation height seismic lines would decrease in landscapes with relatively high densities of anthropogenic features because selection of low vegetation height seismic lines would be diluted within areas where linear features are more common.

## Materials and methods

### Study site

**Figure 1:**
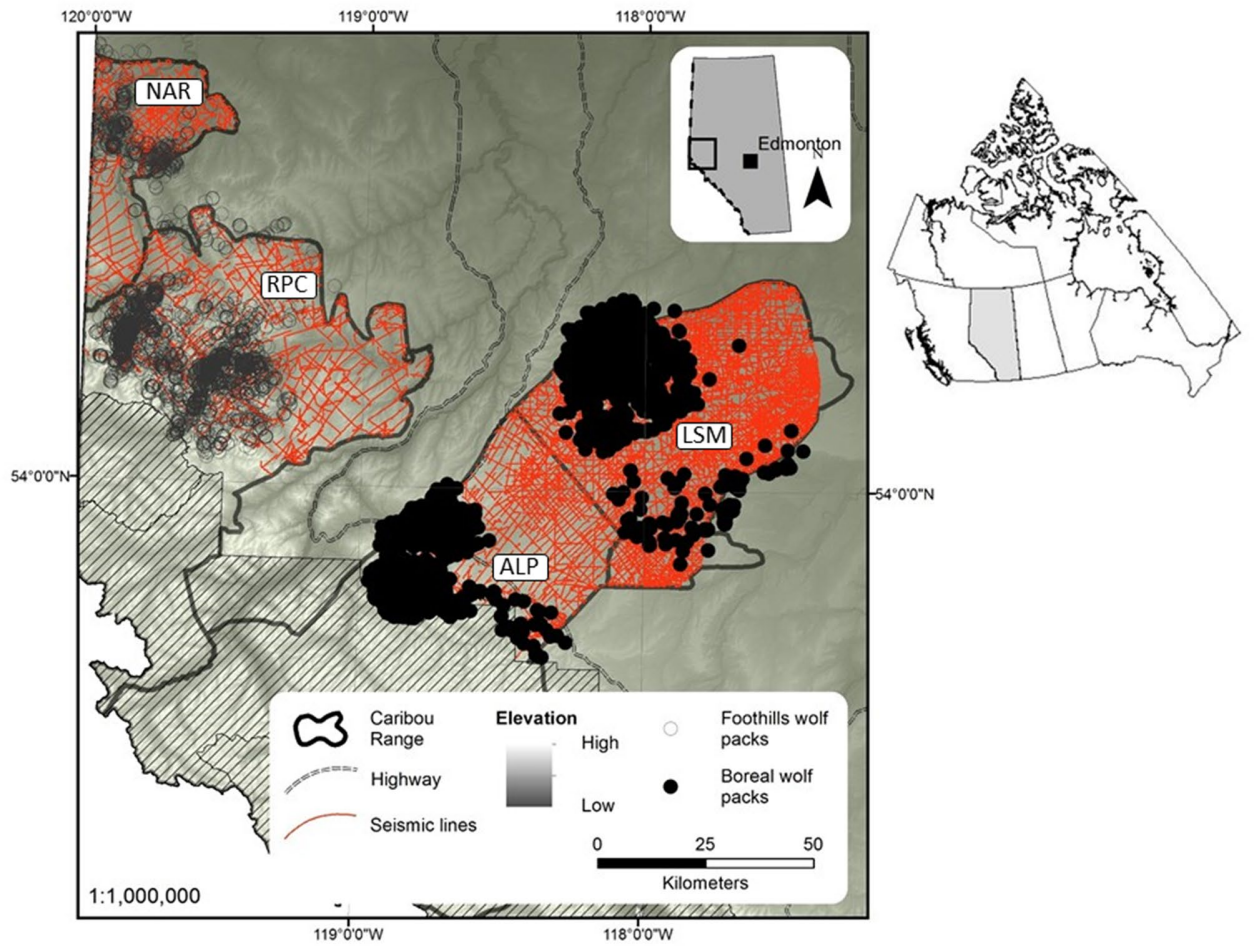
Overview of the more-industrialized boreal landscape and the less-industrialized foothills landscapes in west-central Alberta, Canada. The more-industrialized boreal landscape is delineated by the A La Peche (ALP) and Little Smoky (LSM) caribou ranges, while the less-industrialized foothills landscape is delineated by the Redrock-Prairie Creek (RPC) and Narraway (NAR) caribou ranges. Also shown are protected areas (hatched area), elevation gradient, main highways, legacy seismic lines, and locations of wolves collared in both landscapes between 2003 and 2009.

We confined the study area to public lands within the provincial caribou range boundaries of four woodland caribou herds in west-central Alberta: Little Smoky (LSM), A La Peche (ALP), Narraway (NAR), and Redrock-Prairie Creek (RPC; Fig. 1). This area ranges between 650 and 3,320 m in elevation and includes two natural subregions (upper foothills and lower foothills), with 12,981 km^2^ of lands managed by the provincial government. We did not include mountainous portions of the caribou ranges because those areas are largely within protected areas with few anthropogenic features (Fig. 1). We separated the study area into two regions with distinct densities of anthropogenic features (i.e., well sites, roads, pipelines, seismic lines, and harvest blocks): the more-industrialized boreal landscape has a high density of anthropogenic features (0.41 km^2^/km^2^) with an average of 3.35 km/km^2^ of linear features. The less-industrialized foothills landscape has a lower density of anthropogenic features (0.26 km^2^/km^2^) with an average of 1.57 km/km^2^ of linear features. The more-industrialized boreal landscape includes the territories of five wolf packs (A La Peche, Berland, Horse Creek, Muskeg, and Simonette) that fall within the LSM and ALP caribou ranges, and the less-industrialized foothills landscape includes the territories of three wolf packs (Kakwa, Narraway, and Two Lakes) that fall within the RPC and NAR caribou ranges. Although the less-industrialized foothills landscape is more rugged than the boreal landscape, both areas are dominated by forests of lodgepole pine (*Pinus contorta*), white spruce (*Picea glauca*), and black spruce (*Picea mariana*) with patches of trembling aspen (*Populus tremuloides*) and balsam poplar (*Populus balsamifera*) in upland areas, and larch (*Larix laricina*) in low areas. In addition to caribou, ungulates include whitetail and mule deer (*Odocoileus virginianus* and *O. humionus*), moose (*Alces alces*), and elk (*Cervus elaphus*). Predators include grizzly bears (*Ursus arctos*), black bears (*Ursus americanus*), cougars (*Felis concolor*), wolves (*Canis lupus*), lynx (*Lynx canadensis*), wolverines (*Gulo gulo*), and coyotes (*Canis latrans*).

### Telemetry data

We investigated how seismic lines, and seismic line attributes influenced habitat selection patterns for 15 wolves from eight packs using Global Positioning System (GPS) data from wolves collared between 2003 and 2009. Wolf GPS data were collected as part of research by the Universities of Alberta (coauthors F.S. and L.N.), Calgary (M.M.), and Montana (M.H.). Capture and handling protocols are described previously^37,38^, and were approved and carried out in accordance to university animal care protocols (University of Montana Animal Use Protocol 059-09MHWB-122209; University of Calgary Animal Use Protocol BI11R-17; University of Alberta Animal Care Committee Standards 99-69). Wolves were fitted with Lotek 2200/3300 (Lotek Engineering Systems, Newmarket, Ontario, Canada). GPS collars were originally programmed to acquire locations at a range of intervals between 15-min and 2-h, and we rarefied these locations to 2-h intervals to reduce autocorrelation and obtain more uniform sample sizes among individuals. We also partitioned wolf GPS data into “resting-feeding” and “travelling” locations because selection patterns likely differs between hunting, travelling, or searching behaviour and resting-feeding behaviour^11^. We defined resting-feeding locations as any location during which wolves spent at least six hours within a 300 m radius^39^. We also divided each year into three seasons (denning, rendezvous, and nomadic) based on wolf behaviour to account for variations in seasonal selection associated with life history requirements^38,40,41^. For analyses, we discarded individuals with fewer than 20 locations per landscape-season-behaviour classes per year, and accounted for within-pack correlation by removing one of every two GPS locations acquired from different individuals of the same pack when these locations occurred < 200 m apart during the same time interval^10^. The total number of GPS collar locations was 6,243 (less-industrialized foothills landscape: 3,731 locations from 8 individual-year; more-industrialized boreal landscape: 2,512 from 10 individual-year, Appendix S1).

### Environmental variables

Using a 25-m digital elevation model, we derived topographic variables including slope (variable names in italics: *Slope*), elevation (*Elev*), topographic position index (*TPI*^42^), and compound topographic index (*CTI*; terrain wetness^43^). Predominant winds are from the south-west in this region and we therefore separated aspect into three binary variables (*fFlat* = 0°; *fLee* = from NW to E aspect; and *fWind* = from SE to W aspect, i.e., *fLee* represents a categorical variable describing the pixel as either NW- to E-facing (1), or not (0)). We also derived yearly landcover and percent canopy cover (*%CC*) variables from a combination of Moderate Resolution Imaging Spectroradiometer (MODIS) and Landsat imagery mapped at a 30-m resolution^44,45^. We grouped landcover classes into three categories (*fMixed*, *fConifer*, and *fNon-forest*), and recoded these categories into binary variables. We extracted timber harvest block locations and age from Alberta Vegetation Inventory (AVI) data provided by Forest Management Agreement (FMA) holders within the study area (Canadian Forest Products, West Fraser Mills Ltd., and Weyerhaeuser Co. Ltd.) to delineate harvest blocks < 25 years old. We then combined timber harvest data with road, seismic line, well site, and pipeline data provided by the Government of Alberta (GoA), FMA holders, and the Alberta Energy Regulator to calculate the density of anthropogenic features for each year of animal data (2003–2009) using a 70-m (*A70*—local) and 1-km (*A1k*—landscape) radii circular moving window average in ArcGIS 10.2^46^. We chose a 70-m radius to represent a local scale based on findings from DeCesare et al*.*^37^, and a 1-km radius as a conservative estimate of the influence of disturbance features at the landscape scale^20,47^. We also created a binary variable representing early successional forests (*E.Seral*) by merging vegetated, but non-forested landcover classes with AVI harvest blocks data < 25 year old. To represent the non-linear diminishing effect of small and large streams with increasing distances, we used an exponential decay function (1 − exp ^(−0.002 × distance (m))^) as described by Nielsen et al*.*^48^ (large streams: *DistW1m* and small streams: *DistW20k*). Finally, we also generated a raster surface representing the distance to the main highway intersecting the more-industrialized boreal landscape (*DistHWY40*), and a raster surface representing the distance to the nearest seismic line (*Dist*).

### Seismic line variables

We used point cloud LiDAR data collected between 2003 and 2008 to extract the maximum vegetation heights, at a 1-m resolution, for 100 m sections of seismic lines spanning the study area (*VegHT*). Details of LiDAR-based extractions of vegetation heights on seismic lines are described previously^10^. We derived seismic line wetness (*WAM*) under each 100 m section of seismic line from the average depth-to-water values extracted from wet areas mapping^49^, and used an exponential decay function (1 − exp^−1.55×WAM(m)^) to rapidly decrease the effect of depth to water at depths > 2 m, and to set values > 3 m as constant because the mean root depth of boreal forest vegetation is 2 ± 0.3 m^50^. Finally, we used Geospatial Modeling Environment (GME)^51^ to determine the landcover category that intersected each 100 m segment of seismic line. When seismic line segments fell within two or more landcover types, we used the majority landcover type along the seismic line section.

### Statistical analyses

Our goal was to understand how wolves, on average, respond to attributes of seismic lines, and whether the density of anthropogenic features surrounding seismic lines leads to scale-specific functional responses in habitat selection. For this, we developed within-home range (3rd order) resource selection functions (RSFs) for each individual and used the inverse of the variance associated with each coefficient to calculate weighted averages—i.e., we calculated population averages from individual-based models giving more weight to coefficients derived from animals with more precise estimates^52^. Before generating individual-based models, we first built population-level baseline generalized linear models (GLMs) for ‘resting-feeding’ and ‘travelling’ locations in each landscape. We generated these baseline GLMs as a tool to identify environmental variables that should be included in all individual-based models to avoid the potential for unreliable interpretations associated with averaging across models built with different sets of baseline variables. Using this approach, we included non-informative variables in individual models but performed model-averaging on a consistent set of variables for each individual within each landscape-season-behaviour class. To optimize model fit, we only retained variables that were influential at the population-level (coefficients from weighted averages that do not overlap zero) for each landscape-behaviour dataset. Collinearity and correlation between variables differed per season and resulted in different baseline models per season within each landscape (Appendix S2). Using these baseline models (M1) and null models (M0) as starting points, we generated 13 population-level GLMs based on a priori consideration of multiple working hypotheses to consider as candidate models for our final individual-based models^53^ (Table 1). We used interaction terms between densities of anthropogenic features at the local (*A70*) and landscape (*A1k*) scales (i.e., measures of changing habitat availability) and the distance to the nearest seismic lines (*Dist*) to assess the potential for functional responses in habitat selection driven by changes in availability of anthropogenic habitat. We expected the influence of anthropogenic features on habitat selection patterns to decrease rapidly and non-linearly with increasing distance between individuals and targeted anthropogenic features^54^. We therefore evaluated two decay functions for the distance-to-the nearest seismic line variable (*Dist*) derived from methods described in Nielsen et al*.*^48^ (1 − exp ^(−0.002 × distance (m))^, and 1 − exp ^(−0.001 × distance (m))^), compared univariate models of the *Dist* variable without any decay function to these two decay functions using AIC, and selected the *Dist* variable with the lowest AIC per dataset for subsequent analyses (Appendix S3).

**Table 1:** Candidate models and associated working hypotheses proposed to explain seasonal (denning, rendezvous, and nomadic) selection of areas near regenerating seismic lines for wolves travelling and resting-feeding in a more-industrialized boreal and less-industrialized foothills landscape of west-central Alberta, Canada between 2003 and 2009.

Model	Hypothesis	Model
M0	Null	~
M1	Baseline	~ Base
M2	Distance to seismic lines	~ Base + *Dist*
M3	Distance and landscape functional response	~ Base + Dist*A1k
M4	Distance and local functional response	~ Base + Dist*A70
M5	Ease-of-travel	~ Base + VegHT*Dist
M6	Ease-of-travel and elevation	~ Base + VegHT*Dist*Elev
M7	Ease-of-travel and ruggedness	~ Base + VegHT*Dist*TPI
M8	Wet areas	~ Base + WAM*Dist
M9	Wet areas and high-quality browse	~ Base + WAM*Dist*VegHT
M10	Wet early seral forest	~ Base + WAM*Dist*E.Seral
M11	Vegetation height and landscape functional response	~ Base + VegHT*Dist*A1k
M12	Vegetation height and local functional response	~ Base + VegHT*Dist*A70

*VegHT* vegetation, *WAM* wet areas, *E.Seral* early successional forests, *Elev* elevation, *Dist* distance, densities of anthropogenic features at the local (*A70*) and landscape (*A1k*) scales, and topographic position index (*TPI*). Variables are fully described in “Materials and methods” section. ‘Base’ refers to the suite of variables included in the respective landscape-season-behaviour baseline models for each dataset (See Appendix S2 for details of baseline models).

There is currently no consensus on how to best approach model selection using individual-based models to infer population-level behaviours, however, model selection is straightforward when using a population-level approach^53^. Parameters of a variable of interest can only be estimated from averaging individual-based models if each individual is exposed to a range of environmental conditions associated with that variable, and each individual is exposed to the same variables themselves^55^. When using individual-based models: (1) there is currently no clear consensus on how to average across models with different sets of variables, which can yield ambiguous results, and (2) eliminating individuals that lack exposure to the full set of variables of interest inflates the influence of individuals exposed to the full set of variables when identifying the ‘top models’^56^. Moreover, the choice of adopting individual- or population-level inference depends on specific ecological questions, which in our study were inferences at the population-level^55,57^. We therefore chose to perform model selection using population-level models, but used the inverse-weighted coefficients^52^ of individual-based models for population-level inferences (sensu^58,59^). Appendix S4 further explains our rationale, and Supplementary Table S5 compares the results of model selection performed on population-level models to results from model selection performed on individual-level models.

We derived population averages for animals across each landscape-season-behaviour classes (two landscapes: less-industrialized foothills landscape vs. more-industrialized boreal landscape, three seasons: denning, rendezvous, and nomadic, and two behaviours: resting-feeding, and travelling, for a total of 12 distinct datasets) following a ‘design III’ use-availability approach^53,60^. We used GME^51^ and ArcGIS 10.2.2^46^ to generate 20 random ‘available’ point locations for every used GPS collar location per animal-year-season minimum convex polygon (MCP). Fix rates from GPS collars were < 80% and the number of GPS locations per individual in each dataset varied largely due to missed GPS fixes that over- or under-represented certain individuals (Appendix S1). We therefore accounted for unequal probabilities of obtaining successful GPS collar locations (P_fix_) with changing terrain and habitat by using the weight argument of the svyglm function within the survey package in R to specify observation-specific inverse probability weights for used locations of each individual and held the value of P_fix_ for available locations constant within general linear models^61–64^. We restricted available locations to at least 30 m from one another; the size of the raster pixel used for analyses. We excluded one of two correlated variables (r ≥ 0.5) from any model and also excluded variables with Variance Inflation Factors (VIFs) > 3. We standardized all continuous variables to improve model convergence and used a population-level information-theoretic (IT) approach with Akaike’s Information Criterion (AIC) to assess these variables^53,55^. We carried out data exploration and statistical analyses in R^64,65^. We evaluated model performance using leave-one-out (LOO) cross sample validation; iteratively refitting models to subsets of the data following Matthiopoulos et al.^66^, where mean correlations indicate average model performances, with values closer to 1 (range from 0 to 1) indicating stronger fit. We present model results as beta coefficients (β) ± 95% Confidence Intervals (CI) unless otherwise noted, and to address model selection uncertainty, we report influential interactions for all models with weights of evidence (ω_i_) ≥ 0.1^53^.

## Results

### Baseline models

During all seasons, wolves in both landscapes showed similar baseline selection patterns while resting-feeding and travelling (Appendix S2). Overall, wolves generally selected low elevation (less-industrialized landscape β_x̄_ (LCL_x̄_, UCL_x̄_): − 0.6 (− 0.8, − 0.4); more-industrialized landscape: − 0.3 (− 0.4, − 0.1)), gentle slopes (less-industrialized landscape − 0.3 (− 0.4, 0.07); more-industrialized landscape: − 0.1 (− 0.3, 0.02)), and valley bottoms (less-industrialized landscape CTI: 0.2 (0.1, 0.3), TPI: − 0.4 (− 0.5, − 0.2); more-industrialized landscape CTI: 0.1 (0.08, 0.2), TPI: − 0.2 (− 0.4, − 0.1)), near streams (less-industrialized landscape − 0.3 (− 0.5, − 0.08); more-industrialized landscape: − 0.3 (− 0.4, − 0.1)), in mixed forests (less-industrialized landscape 0.3 (− 0.3, 0.8); more-industrialized landscape: 0.3 (− 0.04, 0.6)) or early successional forests (less-industrialized landscape 0.2 (− 0.3, 0.7); more-industrialized landscape: 0.6 (0.2, 1.0)) with low densities of anthropogenic features at the landscape scale (1-km; less-industrialized landscape (0.06 (− 0.1, 0.2); more-industrialized landscape: − 0.4 (− 0.6, − 0.2)) and high densities of anthropogenic features at the local scale (70-m; less-industrialized landscape (0.02 (− 0.1, 0.2); more-industrialized landscape: 0.04 (− 0.01, 0.2)). In the more-industrialized boreal landscape, travelling wolves also generally selected areas near Highway 40, the only highway traversing the area (− 0.3 (− 0.3, − 0.07)).

### Resting-feeding locations: less-industrialized foothills landscape

**Figure 2:**
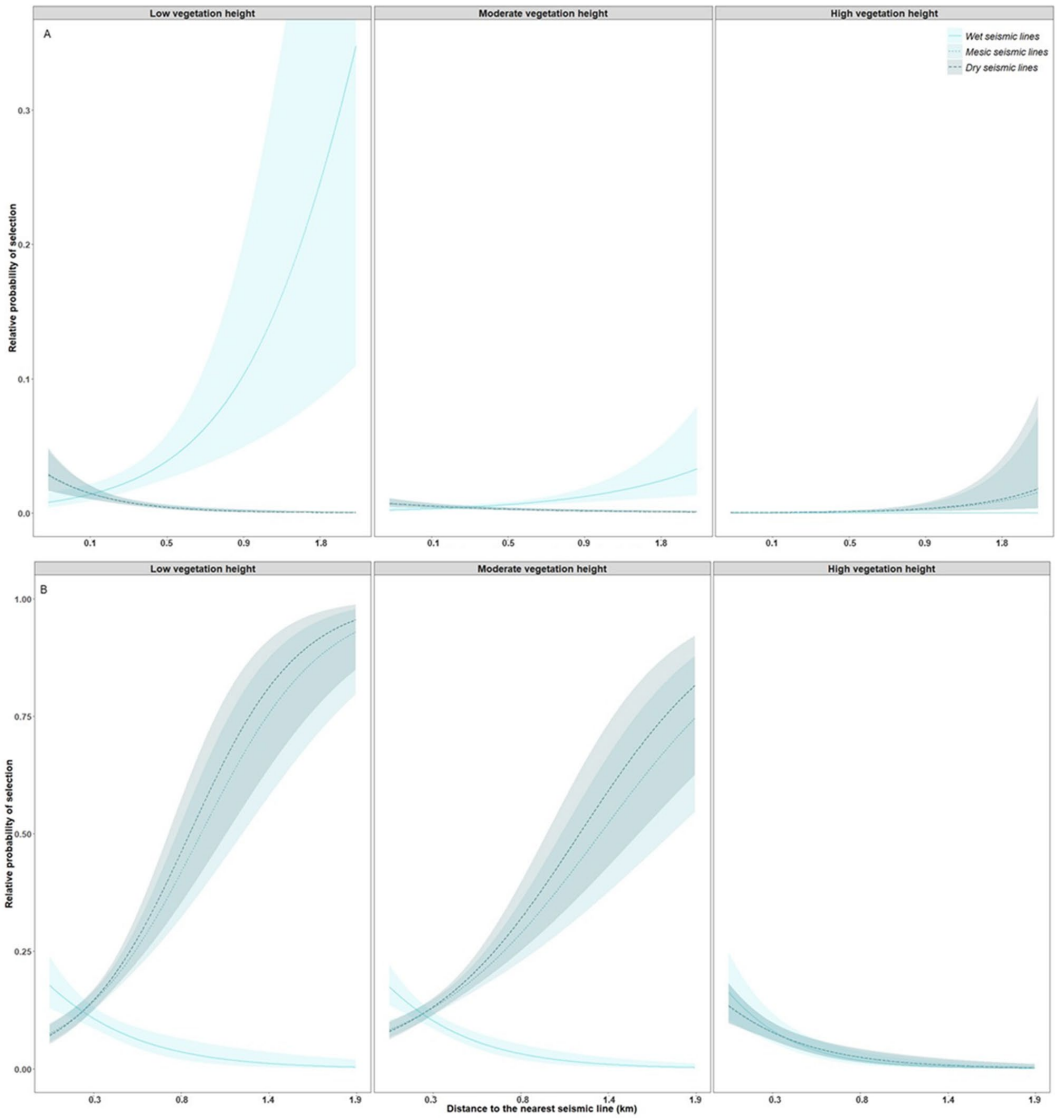
Relative probability of selection from the most-supported models for (**A**) wolves resting or feeding in the less-industrialized foothills landscape during the denning season, and (**B**) wolves resting or feeding in the more-industrialized boreal landscape during the rendezvous season in west-central Alberta, Canada between 2003 and 2009. Shaded areas are 95% prediction intervals. Each predictor variable is plotted within its observed range while other variables are held at their mean. *VegHT* and *WAM* are binned into low, mesic, and high categories based on quantiles for visual interpretation, models were built with continuous variables.

**Figure 3:**
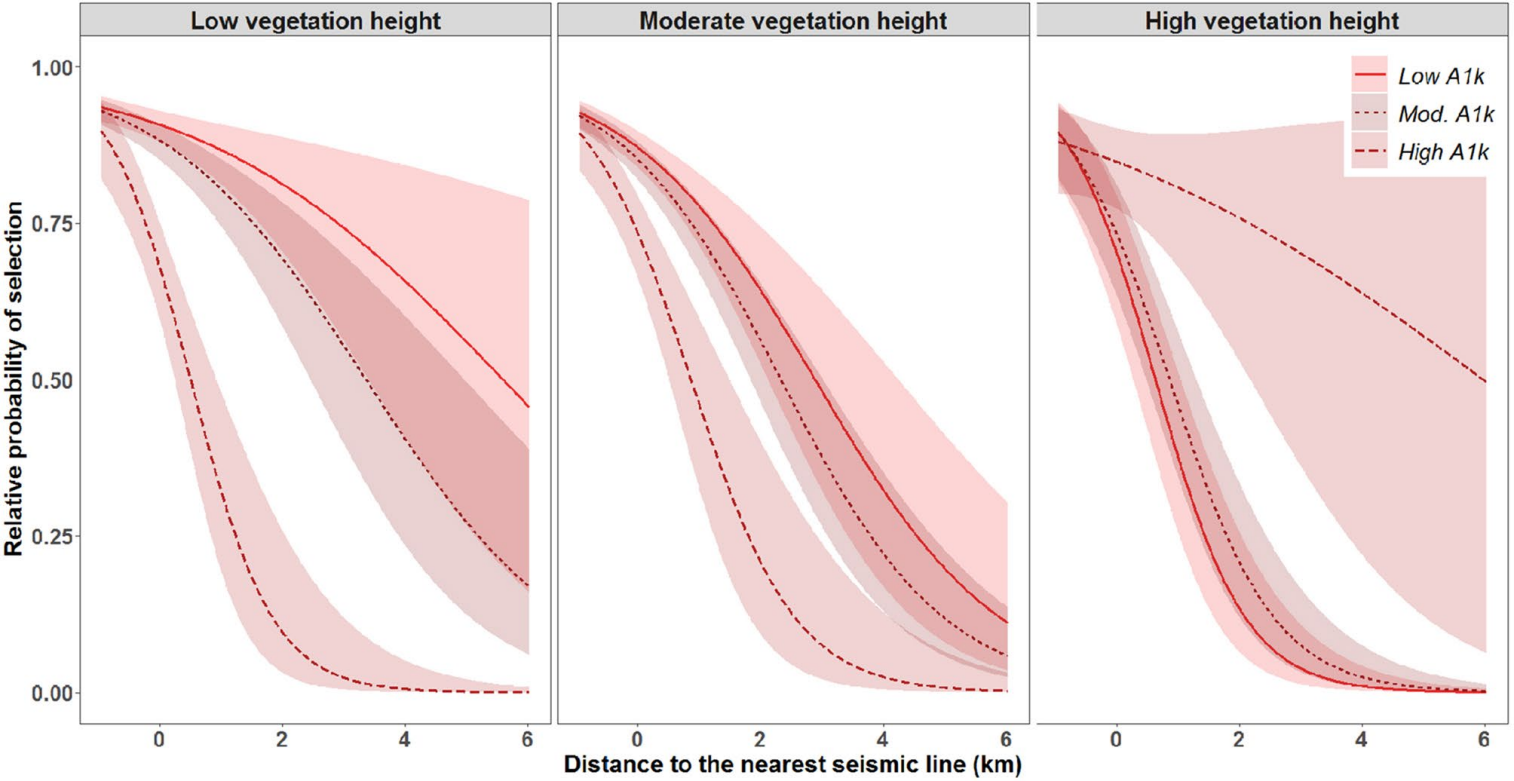
Relative probability of selection from the most-supported models for wolves resting or feeding in the less-industrialized foothills landscape during the rendezvous season in west-central Alberta, Canada between 2003 and 2009. Shaded areas are 95% prediction intervals. Each predictor variable is plotted within its observed range while other variables are held at their mean. *VegHT* and *A1k* are binned into low, moderate, and high categories based on quantiles for visual interpretation, models were built with continuous variables.

Overall, model selection demonstrated high confidence for resting-feeding models. In the less-industrialized foothills landscape, the best selected model was M9: *Wet areas & Browse* for the denning season (ω_i_ > 0.9), M11: *Vegetation height & landscape functional response* for the rendezvous season (ω_i_ > 0.9), and M7: *Ease-of-Travel & Ruggedness* for the nomadic season (ω_i_ > 0.9). During the denning season, wolves selected areas near comparatively dry and low vegetation height seismic lines, while selecting for areas farther from wet, low vegetation height seismic lines, and farther from seismic lines with high vegetation heights, regardless of seismic line wetness (WAM*Dist*VegHT = 0.9 ± 0.05, Fig. 2A, Appendix S5 Table S6). During the rendezvous season, wolves showed evidence of a functional response in habitat selection driven by densities of anthropogenic features at the landscape scale (1-km), where wolves selected areas near comparatively low vegetation height seismic lines more when in areas of low and moderate densities of anthropogenic features (A1k*Dist*VegHT = 0.4 ± 0.05, Fig. 3, Appendix S5 Table S6). Finally, during the nomadic season, wolves selected areas near comparatively low vegetation height seismic lines only when in valley bottoms (low *TPI* values), and increasingly selected areas farther from low vegetation height seismic lines while on ridgetops and hilltops (TPI*Dist*VegHT =  − 0.09 ± 0.01, Appendix S6 Fig. S1, Appendix S7 Table S10 Leave-one-out model validation indicated moderate to high model fit with mean correlations for individuals ranging from 0.4 to 0.7; Appendix S5).

### Resting-feeding locations: more-industrialized boreal landscape

**Figure 4:**
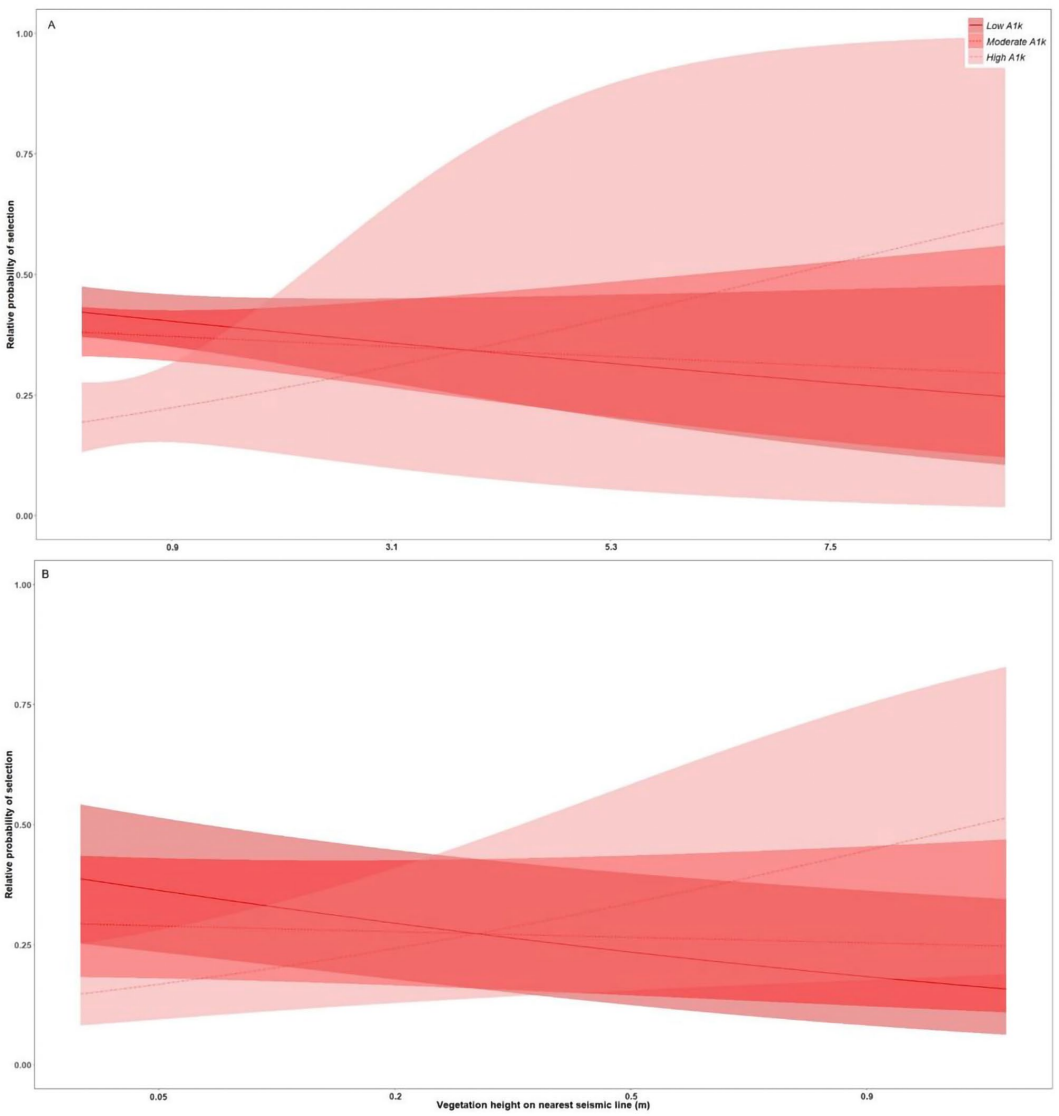
Relative probability of selection from the most-supported models for (**A**) wolves resting or feeding in the more-industrialized boreal landscape during the denning season, and (**B**) wolves resting or feeding in the more-industrialized boreal landscape during the nomadic season in west-central Alberta, Canada between 2003 and 2009. Shaded areas are 95% prediction intervals. Each predictor variable is plotted within its observed range while other variables are held at their mean. *A1k* is binned into low, moderate, and high categories based on quantiles for visual interpretation, models were built with continuous variables.

In the more-industrialized boreal landscape model selection demonstrated moderate to high confidence, and the best selected model was M11: *Vegetation height & landscape functional response* for the denning (ω_i_ > 0.9), and nomadic season (ω_i_ = 0.6), and M9: *Wet areas & Browse* for the rendezvous season (ω_i_ > 0.9). During the denning season, wolves selected areas near low vegetation seismic lines when in areas with low and moderate densities of anthropogenic features at the landscape scale more than when in areas with comparatively higher densities of anthropogenic features (A1k*Dist*VegHT = 0.2 ± 0.07, Fig. 4A, Appendix S5 Table S7). During the rendezvous season, wolves selected areas near relatively wet and low vegetation height seismic lines more than dry, low vegetation height seismic lines, and selected areas near high vegetation seismic lines, regardless of seismic line wetness (WAM*Dist*VegHT = -0.3 ± 0.07, Fig. 2B, Appendix S5 Table S7). Finally, during the nomadic season, we also observed evidence of a functional response in habitat selection driven by the density of anthropogenic features. Wolves selected areas near relatively low vegetation height seismic lines more within areas of low density of anthropogenic features at the landscape scale, and decreased selection for areas near low vegetation height seismic lines more with increasing density of anthropogenic features (A1k*Dist*VegHT = 0.2 ± 0.07, Fig. 4B, Appendix S5 Table S7). Based on the second best model (M6: *Ease-of-Travel & Elevation*, ω_i_ = 0.4), wolves also selected areas near relatively low vegetation height seismic lines compared to high vegetation height seismic lines, and especially selected areas near low and moderate vegetation height seismic lines at low elevations (*Elev* × *VegHT* × *Dist* = 0.2 ± 0.04, Appendix S7 Table S10). Leave-one-out model validation indicated low to moderate model fit with mean correlations for individuals ranging from 0.2 to 0.5; Appendix S5).

### Travelling locations: less-industrialized foothills landscape

Overall, model selection yielded considerable uncertainty when investigating drivers of travelling locations. In the less-industrialized foothills landscape, the best selected model was M6: *Ease-of-Travel & Elevation* for the denning season (ω_i_ = 0.8), M10: *Wet areas & early seral forest* for the rendezvous season (ω_i_ = 0.4), and M8: *Wet areas* for the nomadic season (ω_i_ = 0.5). During the denning season, wolves selected areas near seismic lines at low elevation (Elev*Dist = 0.3 ± 0.08, Appendix S1 Table S3). During the rendezvous season, wolves selected areas near relatively wet seismic lines more than dry seismic lines (WAM*Dist = 0.1 ± 0.08, Appendix S5 Table S8). Based on the next best models (M6: *Ease-of-Travel & Elevation*, ω_i_ = 0.3), these wolves also selected areas near relatively low vegetation height seismic lines compared to high vegetation height seismic lines, and especially selected areas near low and moderate vegetation height seismic lines at low elevations (Elev*VegHT*Dist = 0.1 ± 0.08, Appendix S7 Table S10). During the nomadic season, wolves generally selected areas near relatively dry seismic lines compared to wet seismic lines (WAM*Dist =  − 0.1 ± 0.02, Appendix S1 Table S3), and this result was also supported by the next best model (M10: *Wet areas & early seral forest* for the rendezvous season, ω_i_ = 0.3; WAM*Dist =  − 0.1 ± 0.03, Appendix S7 Table S10). We could not assess model fit for the denning season because of low sample size but leave-one-out model validation for the rendezvous and nomadic seasons indicated moderate to high model fit with mean correlations for individuals ranging from 0.4 to 0.6; Appendix S5).

### Travelling locations: more-industrialized boreal landscape

Overall, model selection yielded considerable uncertainty when investigating drivers of travelling locations, and the best selected model was M2: *Distance to seismic lines* for the denning season (ω_i_ = 0.3), M8: *Wet areas* for the rendezvous season (ω_i_ = 0.3), and M10: *Wet areas & early seral forest* for the nomadic season (ω_i_ = 0.5). During the denning season, wolves generally selected areas farther from seismic lines (*Dist* = 0.2 ± 0.07, Appendix S5 Table S9). However, based on the next best model (M6: *Ease-of-Travel & Elevation*, ω_i_ = 0.1), these wolves also selected areas near low and moderate vegetation height seismic lines more at low elevations (Elev*Dist*VegHT = 0.3 ± 0.06, Appendix S7 Table S10). During the rendezvous season, wolves selected areas near wet seismic lines more than dry seismic lines (WAM*Dist = 0.2 ± 0.09, Appendix S5 Table S9). During the nomadic season, wolves selected areas near wet seismic lines in early successional forests more than areas near dry seismic lines, and also selected areas near wet seismic lines in early successional forests more than areas near seismic lines in mature forests regardless of seismic line wetness (WAM*Dist*E.Seral = 0.7 ± 0.5, Appendix S5 Table S9). Finally, based on the next best model (M4: *Distance & local functional response*, ω_i_ = 0.2), these wolves also selected areas near seismic lines when in areas with high densities of anthropogenic features at the local scale (A70*Dist = − 0.3 ± 0.2, Appendix S7 Table S10). Leave-one-out model validation indicated moderate model fit with mean correlations for individuals ranging from 0.4 to 0.5; Appendix S5).

## Discussion

Using GPS collar data spanning 7 years and collected from eight resident wolf packs in west-central Alberta, Canada, we demonstrated evidence of consistent landscape-scale functional response in habitat selection driven by industrial activity, where wolves were more likely to rest or feed in areas near relatively low vegetation height seismic lines within areas of low densities of anthropogenic features. Overall, we confirmed seasonal and landscape-based behavioural differences that (a) support evidence that seismic lines facilitate ease-of-travel^9,10^, especially in landscapes with comparatively low densities of anthropogenic features, and during the denning season when adults travel long distances to hunt, patrol the territory, and return to dens, and (b) that seismic lines potentially facilitate search for prey species while pup rearing during the snow-free rendezvous season when wolves typically select wet meadows^10,11,31^, especially within landscapes with comparatively high densities of anthropogenic features. Overall, our results suggest that effects of industrial activity drive a functional response in habitat selection for wolves selecting areas near seismic lines. Although seasonal patterns of selection were generally consistent for wolves across west-central Alberta, Canada, landscape heterogeneity highlights the usefulness of seismic lines for travel within landscapes with low densities of anthropogenic features, and the potential benefits of seismic lines towards search for primary prey within landscapes with comparatively high densities of anthropogenic features.

Our study is unique because although the cumulative effects of industrial activity have been investigated previously for wolves (e.g.^23,24^), our study is the first to specifically observe evidence of (1) behaviour-specific (i.e., resting-feeding vs. travelling) responses to variation in vegetation height on seismic lines, and (2) year-round landscape-scale functional response in habitat selection in response to variation in vegetation height on seismic lines. In two distinct landscapes with largely dissimilar densities of anthropogenic features, we found that wolves consistently selected for seismic lines with relatively low vegetation height when in areas of low densities of anthropogenic features. These results support evidence that seismic lines covered with lower vegetation are likely more useful to wolves for travel and search for prey compared to seismic lines with high vegetation^9,10,19^, and especially in areas where fewer of these linear features are available on the landscape. Although seismic lines in both landscapes are utilized by humans using motorized Off-Highway Vehicles (OHV^15^), human activity on seismic lines is likely not a major deterrent for wolves using seismic lines in either landscapes because unlike frequent vehicular traffic observed on roads^11,24^, the frequency of OHV traffic on seismic lines is minimal. We therefore propose that the functional response in habitat selection observed here is likely a result of seismic lines enhancing ease-of-travel or potentially, search for prey, and that with increasing densities of anthropogenic features, the enhanced value of individual linear features to wolves decreases, and therefore dilutes the overall wolf response, especially within areas where seismic lines are pervasive.

In accordance with previous studies, e.g.^24,67^, we observed evidence of functional response in habitat selection driven by densities of anthropogenic features primarily during the snow-free seasons, although wolves in the more-industrialized boreal landscape were also more likely to select areas near relatively low vegetation height seismic lines during the nomadic season. Although we were unable to evaluate the effect of snow depth and snow compaction on wolf behaviour in our study, Droghini and Boutin^68^ recently observed that snow compaction from motorized vehicle use of linear features reduced wolf sinking depth, and therefore likely favors travel efficiency. Results from Droghini and Boutin^68^ therefore offer a potential explanation for the selection of areas near relatively low vegetation heights seismic lines during winter in the more-industrialized boreal landscape, and for the generally low model fit that we observed with the nomadic season in this landscape.

Within the less-industrialized foothills landscape, wolf selection patterns relative to seismic lines were generally in line with previous evidence that seismic lines facilitate travel, potentially because the relatively low densities of anthropogenic features in this landscape amplifies the usefulness of individual linear features. Whittington et al*.*^11^ observed that wolves increasingly selected for linear features at high elevation, and here, we observed that wolves travelling in the less-industrialized foothills landscape increasingly selected for relatively low vegetation height seismic lines when travelling at low elevation. It is likely that relatively low vegetation seismic lines at low elevation and in more rugged terrain below treeline facilitate wolf travel compared to seismic lines with high vegetation or compared to seismic lines above treeline where vegetation is generally sparse. Wolves resting or feeding in the less-industrialized foothills landscape generally selected dry seismic lines, and these results are consistent with selection of seismic lines to improve ease-of-travel when prey are more diffuse, and when adults periodically return to dens or central locations^28,40^. Looking at broad-scale travelling locations within west-central Alberta, Finnegan et al*.*^10^ also observed that wolves moved towards seismic lines with low vegetation heights more during the denning and rendezvous seasons, and especially towards low vegetation seismic lines in non-mature forests compared to higher vegetation seismic lines in mixed and conifer forests. Finnegan et al.^10^ also observed that wolves stepped towards high vegetation height seismic lines during the denning season, and our analysis investigating functional responses in selection of seismic lines while accounting for landscape heterogeneity, further explains the findings of Finnegan et al.^10^. Specifically, we observed higher selection for low vegetation height seismic lines within landscapes with low and moderate densities of anthropogenic features, and increased selection for areas near high vegetation height seismic lines with increasing densities of anthropogenic features. We therefore highlight the need to consider landscape heterogeneity and functional response when investigating animal behaviour. In addition, consistent with patterns observed in the more-industrialized boreal landscape and habitat favoring high-quality browse species preferred by primary prey^2,10,69^, we observed that wolves travelling within the less-industrialized landscape during the rendezvous season also selected areas near wet seismic lines in early successional forests, indicating that although seismic lines are likely used to improve ease-of-travel, wolves in the less-industrialized landscape may also be targeting seismic lines in areas potentially preferred by moose, deer, and elk while pups aren’t able to travel long distances.

Within the more-industrialized boreal landscape, wolf selection patterns relative to seismic lines generally supported behaviour more in line with searching for primary prey, potentially because high densities of anthropogenic features available in this landscape dilutes the usefulness of linear features for ease-of-travel. Consistent with habitats likely preferred by primary prey^35,36^, and with previous observations that wolves may opt to increase hiding cover and prey availability for pups during the rendezvous season^31^, wolves resting, feeding, or travelling in the more-industrialized boreal landscape during the rendezvous season selected relatively wet and low vegetation height seismic lines, and also selected relatively high vegetation height seismic lines regardless of seismic line wetness. Interestingly, selection patterns during the denning season, when adults may travel long distances to find prey while having to return to dens to feed pups, were more consistent with observations from the less-industrialized landscape where wolf selection supported ease-of-travel. It is plausible that because the more-industrialized boreal landscape has a high overall density of anthropogenic features, the usefulness of seismic lines to improve ease-of-travel is only apparent when travel efficiency is most needed, such as during the denning season. During the nomadic season, selection patterns for wolves resting or feeding were also more consistent with observations from the less-industrialized landscapes where wolf selection supported ease-of-travel. However, wolves travelling in the more-industrialized boreal landscape during the nomadic season were again consistent with areas associated with high-quality browse species for primary prey as we observed selection for areas near relatively wet seismic lines in early successional forests, and no selection for areas near seismic lines within mature stands^29,36^.

Our results highlight the importance of considering landscape heterogeneity and habitat characteristics, effects of industrial activity, and the functional response in habitat selection when investigating seasonal behaviour-based selection patterns. This is especially true in landscapes where restoration activity could influence predator–prey dynamics that have implications for management and recovery action affecting threatened species. We propose that by considering the spatial composition and arrangement of landscape features (i.e., spatial heterogeneity), when developing time-sensitive restoration actions, land managers have the ability to alter predator–prey dynamics. For example, to potentially avoid heightening wolf selection for linear features that remain unrestored within an otherwise restored area, land managers would need to consider the density of remaining anthropogenic features, the vegetation height on remaining linear features, and the potential for additional anthropogenic features with future resource extraction. By ensuring that restored areas do not confine and redirect wolves onto remaining attractive linear features that could improve travel efficiency for wolves (i.e., low vegetation seismic lines within areas of low densities of anthropogenic features at the landscape scale), land managers could potentially reduce predation risk for threatened prey species.

The results of this research offer unique insights into the effects of specific management actions on the functional response in habitat selection, and ultimately, on predator–prey dynamics in relation to landscape-specific spatial heterogeneity. Our results can help managers prioritize actions aimed towards the recovery of threatened species such as caribou by ensuring that restored areas do not heighten wolf selection of remaining and attractive linear features such as low vegetation seismic lines in areas with low densities of anthropogenic features. Restoring anthropogenic features that are most selected by predators, and that therefore likely facilitate hunting efficiency^11,70^, can decrease the potential overlap between predator and prey, but may also increase the selection of remaining seismic lines by predators (i.e., increase the strength of habitat-driven functional responses for predators on remaining and attractive anthropogenic features), therefore potentially heightening predation risk in unrestored areas. With the goal of effective landscape restoration in mind, we propose the need for management actions that consider the potential ripple effects of specific human activity (restoration or continued industrial activity) on predator–prey dynamics.

## Supplementary information

Supplementary Information

## Data Availability

The datasets generated during and/or analysed during the current study are available in the Dryad repository (https://doi.org/10.5061/dryad.djh9w0vxd).
