# Supplementary material for: The density of anthropogenic features explains seasonal and behaviour-based functional responses in selection of linear features by a social predator
Source: Sci Rep. 2020 Jul 10;10:11437. doi: 10.1038/s41598-020-68151-7 (PMC7351780; doi:10.1038/s41598-020-68151-7)
Supplement: Supplementary file 1 — Supplementary Information [file 41598_2020_68151_MOESM1_ESM.pdf]

Supporting Information

Title: *The density of anthropogenic features explains seasonal and behaviour-based functional responses in selection of linear features by a social predator*

Pigeon, E. Karine[1,2*] (Karine.pigeon@gmail.com), D. MacNearney[1,3], M. Hebblewhite[4], M. Musiani[5], L. Neufeld[6], J. Cranston[7], G. Stenhouse[1], F. Schmiegelow[8], and L. Finnegan[1]

[1*]fRI Research, 1176 Switzer Drive, Hinton, Alberta, Canada

[2]Current address: Geomatics and Landscape Ecology Laboratory (GLEL), Carleton University, Ottawa, Canada

[3]Current address: Wildlife Research Division, Environment and Climate Change Canada, National Wildlife Research Centre, Ottawa, Ontario, Canada

[4]Department of Ecosystem and Conservation Science, W.A. Franke College of Forestry and Conservation, University of Montana, Missoula, Montana, USA

[5]Department of Biological Sciences, Faculty of Science, University of Calgary, Calgary, Alberta, Canada

[6]Parks Canada, Jasper National Park, Jasper, Alberta, Canada

[7]Arctos Ecological Consultants, Edmonton, Alberta, Canada

[8]Department of Renewable Resources, University of Alberta, Edmonton, Alberta and Yukon Research Centre, Yukon University, Whitehorse, Yukon, Canada

Appendix S1

Table S1. Percentage of successful GPS locations acquired (Fix rate) per individual in (A) the more industrialized landscape and (B) the less industrialized landscape across seasons (denning, nomadic, and rendezvous) for travelling and resting-feeding behaviour used to investigate seasonal wolf selection near regenerating seismic lines in west-central Alberta, Canada between 2003 – 2009.

More industrialized		Less industrialized	
Individual ID	Fix rate	Individual ID	Fix rate
W36_2003	0.65	W105_2008	0.63
W37_2003	0.73	W110_2007	*0.55*
W37_2004	0.64	W116_2007	*0.62*
W43_2004	0.51	W124_2008	*0.52*
W46_2004	0.74	W124_2009	*0.77*
W47_2004	0.74	W126_2008	*0.59*
W48_2004	0.78	W127_2008	*0.69*
W114_2007	0.52	W127_2009	*0.79*
W119_2007	0.37	-	-
W122_2009	0.50	-	-
Range 0.41 (0.37 to 0.78)		*Range: 0.2 (0.52 to 0.79)*	

Appendix S2

Table S2. Parameter estimates (β) and 95% confidence intervals (LCI, UCI) for travelling (A) and resting – feeding (B) baseline models investigating seasonal (denning, nomadic, and rendezvous) selection from 8 individual-wolf-year near regenerating seismic lines in the less-industrialized foothills landscape of west-central Alberta, Canada between 2003 – 2009. We only retained variables that were influential at the population-level for each landscape-behaviour dataset, but collinearity and correlation between variables differed per season and resulted in different baseline models per season-behaviour-landscapes. Influential variables are shown in bold, and variables are fully described in *Materials and Methods*.

(A)	β	LCI	UCI	(B)	β	LCI	UCI
Denning							
**Intercept**	**-3.2**	**-3.5**	**-2.8**	**Intercept**	**-3.7**	**-4.0**	**-3.3**
E.Seral	0.5	-0.1	1.1	E.Seral	-0.3	-0.9	0.3
fFlat	-0.7	-1.4	0.03	**fLee**	**-0.6**	**-0.8**	**-0.3**
**fLee**	**-0.8**	**-1.1**	**-0.4**	**fMixed**	**0.7**	**0.4**	**1.0**
fMixed	0.5	-0.00003	0.9	**A1k**	**0.4**	**0.3**	**0.6**
A1k	0.08	-0.1	0.3	CC	-0.08	-0.2	0.03
**CC**	**-0.2**	**-0.4**	**-0.04**	CTI	-0.03	-0.1	0.1
**CTI**	**0.2**	**0.03**	**0.3**	DistHwy40	-0.01	-0.2	0.2
**Elev**	**-1.0**	**-1.3**	**-0.7**	**Elev**	**-1.5**	**-1.9**	**-1.2**
A70	0.04	-0.1	0.2	**A70**	**-0.3**	**-0.5**	**0.1**
**Slope**	**-0.4**	**-0.6**	**-0.1**	**Slope**	**-0.2**	**-0.4**	**-0.06**
Rendezvous							
**Intercept**	**-3.0**	**-3.4**	**-2.7**	**Intercept**	**-3.1**	**-3.4**	**-2.8**
E.Seral	0.06	-0.7	0.6	**E.Seral**	**0.6**	**0.05**	**1.0**
fFlat	-2.2	-4.4	0.09	fLee	-0.1	-0.5	0.3
fLee	0.01	-0.4	0.4	fMixed	-0.009	-0.7	0.7
fMixed	0.2	-0.5	0.9	A1k	-0.03	-0.2	0.2
A1k	-0.09	-0.3	0.1	**DistW1m**	**-0.3**	**-0.6**	**-0.06**
**CC**	**-0.5**	**-0.7**	**-0.3**	**CTI**	**0.2**	**0.05**	**0.3**
**CTI**	**0.3**	**0.1**	**0.4**	**Elev**	**-0.2**	**-0.5**	**-0.02**
**Elev**	**-0.6**	**-0.9**	**-0.3**	**A70**	**0.3**	**0.1**	**0.4**
A70	0.1	-0.02	0.3	**Slope**	**-0.3**	**-0.6**	**-0.08**
**Slope**	**-0.3**	**-0.5**	**-0.07**	**TPI**	**-0.6**	**-0.9**	**-0.3**
Nomadic							
**Intercept**	**-2.9**	**-3.0**	**-2.7**	**Intercept**	**-3.0**	**-3.1**	**-2.9**
**E.Seral**	**0.4**	**0.06**	**0.7**	E.Seral	0.04	-0.1	0.2
fFlat	0.1	-0.4	0.6	**fLee**	**-0.1**	**-0.2**	**-0.002**
fLee	-0.1	-0.3	0.08	**fMixed**	**0.4**	**0.3**	**0.6**

**fMixed**	**0.3**	**0.00002**	**0.6**	**A1k**	**0.1**	**0.09**	**0.2**
**A1k**	**-0.1**	**-0.2**	**-0.03**	**CTI**	**0.3**	**0.3**	**0.4**
**A70**	**0.2**	**0.2**	**0.3**	**DistW1m**	**-0.3**	**-0.4**	**-0.3**
**CTI**	**0.3**	**0.2**	**0.4**	**Elev**	**-0.3**	**-0.3**	**-0.2**
**DistW1m**	**-0.2**	**-0.3**	**-0.1**	**A70**	**-0.2**	**-0.2**	**-0.09**
Elev	-0.03	-0.1	0.1	**Slope**	**-0.2**	**-0.2**	**-0.1**
Slope	-0.05	-0.2	0.05	**TPI**	**-0.3**	**-0.4**	**-0.2**
**TPI**	**-0.2**	**-0.3**	**-0.1**	DistHwy40	0.005	-0.04	0.05

Table S3. Parameter estimates (β) and 95% confidence intervals (LCI, UCI) for travelling (A) and resting – feeding (B) baseline models investigating seasonal (denning, nomadic, and rendezvous) selection from 12 individual-wolf-year near regenerating seismic lines in the more-industrialized boreal landscape of west-central Alberta, Canada between 2003 – 2009. We only retained variables that were influential at the population-level for each landscape-behavoiur dataset but collinearity and correlation between variables differed per season and resulted in different baseline models per season-behaviour-landscapes. Influential variables are shown in bold, and variables are fully described in *Materials and Methods.*

(A)	β	LCI	UCI	(B)	β	LCI	UCI
Denning							
**Intercept**	**-3.3**	**-3.6**	**-3.1**	**Intercept**	**-3.9**	**-4.07**	**-3.7**
E.Seral	0.4	-0.1	0.9	**E.Seral**	**0.7**	**0.4**	**1.0**
fFlat	0.3	-0.4	0.9	**fLee**	**0.7**	**0.5**	**0.9**
fLee	0.2	-0.1	0.5	fMixed	-0.2	-0.4	0.07
fMixed	-0.05	-0.4	0.3	A70	-0.2	-0.3	0.03
**A1k**	**-0.5**	**-0.7**	**-0.2**	**CTI**	**0.1**	**0.06**	**0.2**
A70	0.1	-0.1	0.3	**DistW1m**	**-0.8**	**-0.9**	**-0.6**
**CTI**	**0.3**	**0.2**	**0.4**	DistHwy40	-0.07	-0.1	0.001
**DistW1m**	**-0.4**	**-0.5**	**-0.2**	**Elev**	**-0.4**	**-0.5**	**-0.3**
**DistW20k**	**-0.2**	**-0.4**	**-0.03**	Slope	0.05	-0.07	0.2
**Elev**	**-0.2**	**-0.3**	**-0.02**	**TPI**	**-0.4**	**-0.5**	**-0.4**
Slope	-0.1	-0.2	0.1				
**TPI**	**-0.2**	**-0.4**	**-0.08**				
Rendezvous							
**Intercept**	**-2.9**	**-3.1**	**-2.7**	**Intercept**	**-3.3**	**-3.4**	**-3.1**
**E.Seral**	**0.5**	**0.2**	**0.8**	**E.Seral**	**0.5**	**0.2**	**0.8**
fFlat	-0.2	-0.8	0.3	**fLee**	**-0.2**	**-0.4**	**-0.06**
**fLee**	**-0.5**	**-0.7**	**-0.3**	**fMixed**	**0.3**	**0.04**	**0.5**
**fMixed**	**0.01**	**-0.3**	**0.4**	**A1k**	**-0.5**	**-0.6**	**-0.4**
**A1k**	**-0.3**	**-0.4**	**-0.1**	A70	-0.06	-0.1	0.1
**A70**	**0.2**	**0.1**	**0.3**	CTI	-0.03	-0.1	0.05
**CTI**	**0.1**	**0.04**	**0.2**	**DistHwy40**	**-0.2**	**-0.3**	**-0.1**
**DistW1m**	**-0.3**	**-0.4**	**-0.1**	DistW1m	-0.01	-0.1	0.1
DistW20k	-0.2	-0.3	-0.005	**Elev**	**-0.2**	**-0.3**	**-0.08**
**Elev**	**-0.2**	**-0.4**	**-0.1**	**Slope**	**-0.5**	**-0.7**	**-0.4**
**Slope**	**-0.2**	**-0.4**	**-0.05**	**TPI**	**-0.3**	**-0.4**	**-0.3**
TPI	-0.06	-0.2	0.09				
Nomadic							
**Intercept**	**-3.6**	**-4.0**	**-3.3**	**Intercept**	**-3.4**	**-3.6**	**-3.2**
**E.Seral**	**1.1**	**0.6**	**1.6**	**E.Seral**	**0.4**	**0.1**	**0.7**

fFlat	-0.6	-1.5	0.3	fLee	0.03	-0.2	0.2
**fLee**	**-0.8**	**-1.2**	**-0.5**	**fMixed**	**0.4**	**0.1**	**0.6**
**fMixed**	**1.2**	**0.7**	**1.7**	**A1k**	**-0.5**	**-0.6**	**-0.4**
A1k	-0.2	-0.5	0.05	A70	0.01	-0.1	0.2
**A70**	**0.2**	**0.03**	**0.3**	**CTI**	**0.2**	**0.2**	**0.3**
**CTI**	**0.2**	**0.05**	**0.3**	**DistHwy40**	**-0.5**	**-0.6**	**-0.3**
**DistW1m**	**-0.4**	**-0.6**	**-0.2**	**DistW1m**	**0.2**	**0.1**	**0.3**
**DistW20k**	**-0.6**	**-0.9**	**-0.3**	**Elev**	**-0.6**	**-0.7**	**-0.5**
Elev	0.1	-0.06	0.3	**Slope**	**0.2**	**0.1**	**0.3**
**Slope**	**-0.2**	**-0.5**	**-0.007**	**TPI**	**-0.4**	**-0.5**	**-0.2**
**TPI**	**0.08**	**-0.2**	**0.3**				

Appendix S3

Table S4. Decay function for the distance to nearest seismic line (*Dist*) variable in travelling and resting – feeding models investigating seasonal (denning, nomadic, and rendezvous) wolf selection near regenerating seismic lines in west-central Alberta, Canada between 2003 – 2009. Dashes represent datasets where no decay function was used. Decay functions follow the equation described in Nielsen et al. (2009), i.e., 1-exp $^{(-0.001 \times \text{distance (m)})}$ or 1-exp $^{(-0.002 \times \text{distance (m)})}$).

More-industrialized boreal landscape	Decay	Less-industrialized foothills landscape	Decay
Travelling		*Travelling*	
Denning	-0.002	Denning	-0.001
Nomadic	-0.001	Nomadic	-0.002
Rendezvous	-0.001	Rendezvous	-
Resting - feeding		*Resting - feeding*	
Denning	-0.001	Denning	-0.001
Nomadic	-0.002	Nomadic	-
Rendezvous	-	Rendezvous	-

Appendix S4

We ran analyses as a 2-stage approach: We (1) used population-level baseline models as a means to choose which environmental variables should be consistently included in subsequent individual-based models, (2) accounted for GPS fix success rates by individual animals (i.e. instances when the GPS collar should have recorded a location as programed, but didn't), and (3) obtained population-level inferences by averaging parameter estimates from individual-based models across animals weighted by the inverse of the variance to account for unequal sample sizes within animals. While this approach results in the inclusion of some non-informative variables (coefficients that overlapped zero) for some individuals, it also avoids the difficult interpretation and averaging of coefficients from individual models with varying alternative variables. In summary, to obtain population-level inferences, we performed model selection on population-level models and used an individual-based step selection function (SSF) model approach to inform the covariate structure of such population-level inferences.

To our knowledge, accounting for individuals as random effects and correcting for the probability of missed GPS fixes cannot yet be implemented in a binomial GLMM framework (see inverse probability weights in a binomial mixed modelling framework). However, the number of locations acquired per individuals in each landscape-season-behaviour of our datasets varied largely due to missed GPS collar fixes, and these missed fixes over- or under-represents certain individuals (Appendix S1). Our observed fix-rates were low enough to significantly affect habitat modeling (Frair et al. 2014). Therefore, we viewed the significant potential for habitat-induced bias arising due to variable fix-rates as the most important problem to address in development of our statistical models because previous studies clearly demonstrated significant biases arising due to uncorrected fix-rate bias (Frair et al. 2004).

Currently available research evidence suggests that our approach to use population-level models as a tool in the initial stage of model building is a sound approach to derive informative population-level inferences within a 2-step, individual-based model selection framework (e.g. Fieberg et al. 2009, 2010). One of the main drawbacks from using the individual-based approach is that animal-specific parameters of a variable cannot be estimated unless each individual is exposed to environmental conditions associated with that variable. Population-level baseline models allow for the inclusion of a consistent suite of variables as baseline variables for all individuals, and the best approach to select the most relevant variables across all individuals is to perform model selection at the population-level. Although we report the results of these baseline models in general terms in the initial section of the results, we make no inferences from these models. We confirm the validity of our statistical approach by comparing model selection results using individual-based models vs population-level models in Appendix S4, Table S5. Model selection from the 2 approaches yields the same 'top models' for 6 out of 12 models, and these 'top models' are the models with high model fit – hence, the model selection approach appears to have little effect on the broad results: Regardless of the approach, model selection shows high certainty for the same top models (6/12), and considerable uncertainty for the other half.

Model selection is straightforward when using a population-level approach, and there is currently no consensus on how to best approach model selection within the individual-based models framework. Some of the challenges with individual-level model selection that our approach overcomes are: (1) averaging across models with different sets of parameters yields ambiguous results (e.g., how to average across an animal that had no exposure to a particular covariate, is it 0 or NA?), and (2) eliminating individuals that lack exposure to the full set of variables of interest inflates the influence of individuals exposed to the full set of variables when identifying the 'top models'.

Literature cited

Fieberg, J., Rieger, R. H., Zicus, M. C., & Schildcrout, J. S. Regression modelling of correlated data in ecology: subject-specific and population averaged response patterns. *Journal of Applied Ecology*, **46**, 1018-1025 (2009).

Fieberg, J., Matthiopoulos, J., Hebblewhite, M, Boyce, M, & Frair, J. Correlation and studies of habitat selection: problem, red herring, or opportunity? *Philos Trans R Soc Lond B Biol Sci*. **365**, 2233-2244 (2010).

Frair, J. L., S. E. Nielsen, E. H. Merrill, S. R. Lele, M. S. Boyce, R. H. M. Munro, G. B. Stenhouse, and H. L. Beyer. 2004. Removing GPS collar bias in habitat selection studies. Journal of Applied Ecology **41**:201-212.

Table S5: Comparison of best selected models investigating wolf selection near regenerating seismic lines in west-central Alberta, Canada between 2003-2009 based on population models and inverse-weighted averaged individual-based models. Performing model selection on population-level models and averaging model weights across individual-based models yielded identical 'top models' in 6 out of the 12 classes of models. Model selection demonstrated high confidence for models investigating wolves resting-feeding in the less industrialized foothills landscape, inconsistent support for models investigating wolves resting-feeding in the more industrialized boreal landscape and travelling in the less industrialized foothills landscape, and considerable uncertainty for models investigating wolves travelling in the more industrialized boreal landscape regardless of the method used for model selection.

Behaviour-landscape	Season	Population (ω_i)	Individual ($\omega_{\bar{x}i}$)	Hypothesis	Model interactions [*individual model interactions if different*]
Resting-feeding - Less industrialized	Denning	M9 (0.9)	M9 (0.5)	Wet areas & browse	WAM:Dist:VegHT
Resting-feeding - Less industrialized	Rendezvous	M11 (0.9)	M11 (0.9)	Vegetation height & landscape functional response	A1k:VegHT:Dist
Resting-feeding - Less industrialized	Nomadic	M7 (0.9)	M7 (0.4)	Ease-of-travel & ruggedness	TPI:VegHT:Dist
Resting-feeding - More industrialized	Denning	M11 (0.9)	M11 (0.7)	Vegetation height & landscape functional response	A1k:VegHT:Dist
Resting-feeding - More industrialized	Rendezvous	M9 (0.9)	M6 (0.6)	Wet areas & browse [*Ease-of-travel & elevation*]	WAM:Dist:VegHT [*Elev:Dist:VegHT*]
Resting-feeding - More industrialized	Nomadic	M11 (0.6)	M10 (0.6)	Vegetation height & landscape functional response [*Wet early seral forest*]	A1k:VegHT:Dist [*WAM:Dist*]
Travelling - Less industrialized	Denning	M6 (0.8)	M9 (0.5)	Ease-of-travel & elevation [*Wet areas & browse*]	Elev:Dist [*WAM:Dist:VegHT*]
Travelling - Less industrialized	Rendezvous	M10 (0.4)	M10 (0.4)	Wet early seral forest	WAM:Dist:Eseral
Travelling - Less industrialized	Nomadic	M8 (0.5)	M8 (0.5)	Wet areas	WAM:Dist
Travelling - More industrialized	Denning	M2 (0.3)	M12 (0.1) M5 (0.1) M11 (0.1)	Distance [*Vegetation height & local functional response / Ease-of-travel / Vegetation height & landscape functional response*]	Dist [*A70:Dist:VegHT*]
Travelling - More industrialized	Rendezvous	M8 (0.3)	M12 (0.3)	Wet areas [*Vegetation height & local functional response*]	WAM:Dist [*A70:Dist:VegHT*]
Travelling - More industrialized	Nomadic	M10 (0.5)	M1 (0.3)	Wet early seral forest [*Baseline*]	ESeral:WAM:Dist [*Baseline – no interactions*]*

**second-ranked model is identical using both approaches (M4)*

Appendix S5

Table S6. Population-level parameter estimates (β) and 95% confidence intervals (±95%CI) for resting – feeding models investigating seasonal (denning, rendezvous, nomadic) wolf selection near regenerating seismic lines in the less-industrialized foothills landscape of west-central Alberta, Canada between 2003 – 2009. Influential variables are shown in bold. The number of individuals with significant positive (+) and negative (-) coefficients (95%CI not overlapping zero) are also shown. Mean (x̄) and range correlations from Leave-one-out (LOO) cross-validation are in italics. Variables are fully described in Materials and Methods, models and associated hypotheses are described in Table 1. LOO R code: https://figshare.com/articles/Supplement_2_R_code_used_for_wolf_analysis_/3550839

	Denning (n = 3)			Rendezvous (n = 3)			Nomadic (n = 4)		
Variables	β	±95%CI	+|-	β	±95%CI	+|-	β	±95%CI	+|-
E.Seral	-0.2	1.2	0|1	**0.7**	**0.2**	**1|1**	-0.002	6.6	1|1
fLee	**-0.4**	**0.1**	**0|1**	-0.07	0.5	0|0	-0.1	0.03	1|2
fMixed	**0.5**	**0.1**	**2|0**	0.02	7.4	0|0	**0.5**	**0.02**	**3|0**
St.CTIPoint	**-0.08**	**0.07**	**0|2**	**0.4**	**0.01**	**2|0**	**0.3**	**0.002**	**3|0**
Elev	**-1.3**	**0.03**	**0|2**	**-0.3**	**0.09**	**0|2**	**-0.6**	**0.01**	**0|3**
Lin70	**-0.2**	**0.03**	**0|2**	-0.09	0.1	0|1	**-0.2**	**0.01**	**0|3**
Slope	-0.09	0.2	0|1	**-0.3**	**0.05**	**0|1**	**-0.1**	**0.02**	**1|2**
Dist	-1.0	0.03	0|2	-0.3	0.1	1|1	-0.2	0.01	0|3
VegHT	-0.6	0.04	0|2	-0.02	0.6	1|1	0.08	0.02	2|1
A1k	**0.4**	**0.02**	**2|0**	0.03	0.9	1|1	**0.1**	**0.01**	**1|1**
CC	**-0.1**	**0.08**	**0|1**	**-0.2**	**0.04**	**0|2**	-	-	-
DST_HWY40	**-0.3**	**0.08**	**1|1**	-	-	-	**0.2**	**0.02**	**1|1**
WAM	0.5	0.1	2|0	-	-	-	-	-	-
DST_W1M	-	-	-	-	-	-	**-0.1**	**0.02**	**0|2**
TPI	-	-	-	**-0.9**	**0.02**	**0|2**	-0.2	0.01	0|3
VegHT:Dist	-0.4	0.05	0|2	-0.09	0.2	0|1	-0.02	0.08	1|1
WAM:Dist	0.6	0.06	2|0	-	-	-	-	-	-
WAM:VegHT	1.2	0.05	2|0	-	-	-	-	-	-
WAM:Dist:VegHT	**0.9**	**0.05**	**2|0**	-	-	-	-	-	-
A1k:Dist	-	-	-	-0.1	0.3	0|1	-	-	-
A1k:VegHT	-	-	-	0.5	0.02	2|0	-	-	-
A1k:VegHT:Dist	-	-	-	**0.4**	**0.05**	**2|0**	-	-	-
TPI:Dist	-	-	-	-	-	-	0.2	0.003	3|0
St.TPI:St.VegHT	-	-	-	-	-	-	0.01	0.1	1|1
TPI:VegHT:Dist	-	-	-	-	-	-	**-0.09**	**0.01**	**0|2**
x̄ correlation (range)	*0.6 (0.1 - 0.9)*			*0.4 (0.2 – 0.6)*			*0.7 (0.6 - 0.9)*		

Table S7. Population-level parameter estimates (β) and 95% confidence intervals (±95%CI) for resting – feeding models investigating seasonal (denning, rendezvous, nomadic) wolf selection near regenerating seismic lines in the more-industrialized boreal landscape of west-central Alberta, Canada between 2003 – 2009. Influential variables are shown in bold. The number of individuals with significant positive (+) and negative (-) coefficients (95%CI not overlapping zero) are also shown. Mean (x̄) and range correlations from Leave-one-out (LOO) cross-validation are in italics. Variables are fully described in Materials and Methods, models and associated hypotheses are described in Table 1. LOO R code: https://figshare.com/articles/Supplement_2_R_code_used_for_wolf_analysis_/3550839

	**Denning (n = 5)**			**Rendezvous (n = 6)**			**Nomadic (n = 6)**		
Variables	β	±95%CI	+|-	β	±95%CI	+|-	β	±95%CI	+|-
E.Seral	**0.4**	**0.2**	**2|0**	**0.9**	**0.07**	**3|1**	**0.4**	**0.2**	**2|0**
fLee	-0.02	1.02	1|3	**-0.3**	**0.06**	**0|3**	0.04	0.5	2|2
fMixed	**0.4**	**0.08**	**2|1**	**0.2**	**0.2**	**3|1**	**0.4**	**0.07**	**3|0**
St.CTIPoint	**0.3**	**0.01**	**3|0**	0.04	0.1	3|1	**0.2**	**0.02**	**3|1**
Elev	**-0.7**	**0.03**	**0|4**	**-0.5**	**0.1**	**1|3**	**-0.8**	**0.03**	**0|5**
Slope	**0.3**	**0.02**	**3|0**	**-0.4**	**0.03**	**0|4**	**0.3**	**0.02**	**3|1**
Dist	0.07	0.1	1|1	-0.5	0.03	1|3	0.01	0.5	1|2
VegHT	-0.01	0.7	2|2	0.2	0.04	2|3	-0.02	0.3	2|2
A1k	-0.5	0.05	1|3	**-0.3**	**0.03**	**3|1**	**-0.5**	**0.05**	**1|3**
DST_HWY40	**-1.0**	**0.04**	**0|4**	**0.5**	**0.1**	**2|1**	**-1.0**	**0.04**	**0|4**
DST_W1M	**0.3**	**0.03**	**2|1**	**-0.09**	**0.07**	**2|2**	**0.3**	**0.03**	**3|1**
TPI	-0.3	0.03	1|3	**-0.4**	**0.03**	**0|3**	**-0.3**	**0.04**	**2|3**
Lin70	0.05	0.3	1|0	**0.1**	**0.05**	**3|1**	0.05	0.3	1|1
WAM	-	-	-	-0.1	0.06	1|3	-	-	-
VegHT:Dist	0.2	0.05	2|1	0.5	0.02	3|1	0.1	0.06	2|1
A1k:Dist	0.3	0.04	2|1	-	-	-	0.3	0.04	2|0
A1k:VegHT	0.2	0.06	2|0	-	-	-	0.2	0.06	2|0
A1k:VegHT:Dist	**0.2**	**0.07**	**3|1**	-	-	-	**0.2**	**0.07**	**3|1**
WAM:Dist	-	-	-	-0.5	0.02	0|4	-	-	-
WAM:VegHT	-	-	-	-0.09	0.1	1|3	-	-	-
WAM:Dist:VegHT	-	-	-	**-0.3**	**0.07**	**0|2**	-	-	-
x̄ correlation (range)	*0.3 (0.1 – 0.6)*			*0.2 (0.1 - 0.4)*			*0.5 (0.2 - 0.8)*		

Table S8. Population-level parameter estimates (β) and 95% confidence intervals (±95%CI) for travelling models investigating seasonal (denning, rendezvous, nomadic) wolf selection near regenerating seismic lines in the less-industrialized foothills landscape of west-central Alberta, Canada between 2003 – 2009. Influential variables are shown in bold. The number of individuals with significant positive (+) and negative (-) coefficients (95%CI not overlapping zero) are also shown. Mean ($\bar{x}$) and range correlations from Leave-one-out (LOO) cross-validation are in italics. Variables are fully described in Materials and Methods, models and associated hypotheses are described in Table 1. LOO R code: https://figshare.com/articles/Supplement_2_R_code_used_for_wolf_analysis_/3550839

<table>
<tr><th></th><th colspan="3">Denning (n = 2)</th><th colspan="3">Rendezvous (n = 3)</th><th colspan="3">Nomadic (n = 4)</th></tr>
<tr><td>Variables</td><td>β</td><td>±95%CI</td><td>+|-</td><td>β</td><td>±95%CI</td><td>+|-</td><td>β</td><td>±95%CI</td><td>+|-</td></tr>
<tr><td>E.Seral</td><td>0.3</td><td>0.9</td><td>1|0</td><td>-1.4</td><td>0.5</td><td>0|2</td><td>0.1</td><td>0.3</td><td>2|1</td></tr>
<tr><td>fLee</td><td>**-0.8**</td><td>**0.08**</td><td>**0|2**</td><td>-0.09</td><td>0.8</td><td>0|1</td><td>-0.05</td><td>0.2</td><td>0|1</td></tr>
<tr><td>fMixed</td><td>**0.5**</td><td>**0.2**</td><td>**1|0**</td><td>0.2</td><td>1.3</td><td>0|1</td><td>0.4</td><td>0.07</td><td>1|1</td></tr>
<tr><td>CTI</td><td>**0.1**</td><td>**0.08**</td><td>**2|0**</td><td>**0.3**</td><td>**0.04**</td><td>**1|0**</td><td>**0.3**</td><td>**0.01**</td><td>**3|0**</td></tr>
<tr><td>Elev</td><td>-1.1</td><td>0.05</td><td>0|2</td><td>**-0.8**</td><td>**0.05**</td><td>**0|2**</td><td>**-0.08**</td><td>**0.1**</td><td>**0|2**</td></tr>
<tr><td>Lin70</td><td>**0.2**</td><td>**0.08**</td><td>**1|0**</td><td>**0.1**</td><td>**0.1**</td><td>**1|0**</td><td>**0.3**</td><td>**0.01**</td><td>**2|0**</td></tr>
<tr><td>Slope</td><td>**-0.3**</td><td>**0.09**</td><td>**0|1**</td><td>**-0.4**</td><td>**0.08**</td><td>**0|1**</td><td>**-0.1**</td><td>**0.03**</td><td>**0|1**</td></tr>
<tr><td>Dist</td><td>0.3</td><td>0.1</td><td>2|0</td><td>0.3</td><td>0.09</td><td>1|0</td><td>-0.03</td><td>0.1</td><td>0|0</td></tr>
<tr><td>A1k</td><td>0.08</td><td>0.2</td><td>1|0</td><td>0.04</td><td>0.6</td><td>0|0</td><td>**-0.09**</td><td>**0.05**</td><td>**0|1**</td></tr>
<tr><td>fFlat</td><td>**-0.7**</td><td>**0.4**</td><td>**0|2**</td><td>**-9.0**</td><td>**0.2**</td><td>**0|2**</td><td>0.5</td><td>0.21</td><td>1|1</td></tr>
<tr><td>CC</td><td>**-0.2**</td><td>**0.07**</td><td>**0|2**</td><td>**-0.5**</td><td>**0.03**</td><td>**0|2**</td><td>-</td><td>-</td><td>-</td></tr>
<tr><td>VegHT</td><td>-0.3</td><td>0.3</td><td>0|1</td><td>-</td><td>-</td><td>-</td><td>-</td><td>-</td><td>-</td></tr>
<tr><td>WAM</td><td>-</td><td>-</td><td>-</td><td>0.1</td><td>0.1</td><td>1|0</td><td>-0.05</td><td>0.05</td><td>1|2</td></tr>
<tr><td>DST_W1M</td><td>-</td><td>-</td><td>-</td><td>-</td><td>-</td><td>-</td><td>**-0.1**</td><td>**0.03**</td><td>**0|3**</td></tr>
<tr><td>TPI</td><td>-</td><td>-</td><td>-</td><td>-</td><td>-</td><td>-</td><td>**-0.2**</td><td>**0.02**</td><td>**0|2**</td></tr>
<tr><td>VegHT:Dist</td><td>0.1</td><td>0.5</td><td>0|0</td><td>-</td><td>-</td><td>-</td><td></td><td></td><td></td></tr>
<tr><td>Elev:Dist</td><td>**0.3**</td><td>**0.08**</td><td>**2|0**</td><td>-</td><td>-</td><td>-</td><td></td><td></td><td></td></tr>
<tr><td>WAM:Dist</td><td>-</td><td>-</td><td>-</td><td>**0.1**</td><td>**0.08**</td><td>**2|0**</td><td>**-0.1**</td><td>**0.02**</td><td>**0|2**</td></tr>
<tr><td>E.Seral:Dist</td><td>-</td><td>-</td><td>-</td><td>-2.3</td><td>0.5</td><td>0|2</td><td></td><td></td><td></td></tr>
<tr><td>E.Seral:WAM</td><td>-</td><td>-</td><td>-</td><td>-0.8</td><td>1.5</td><td>1|1</td><td></td><td></td><td></td></tr>
<tr><td>E.Seral:Dist:WAM</td><td>-</td><td>-</td><td>-</td><td>-0.3</td><td>10.7</td><td>1|1</td><td></td><td></td><td></td></tr>
<tr><td>*$\bar{x}$ correlation (range)*</td><td colspan="3">*did not converge*</td><td colspan="3">*0.4 (0.1 - 0.7)*</td><td colspan="3">*0.6 (0.5 - 0.8)*</td></tr>
</table>

Table S9. Population-level parameter estimates (β) and 95% confidence intervals (±95%CI) for travelling models investigating seasonal (denning, rendezvous, nomadic) wolf selection near regenerating seismic lines in the more-industrialized boreal landscape of west-central Alberta, Canada between 2003 – 2009. Influential variables are shown in bold. The number of individuals with significant positive (+) and negative (-) coefficients (95%CI not overlapping zero) are also shown. Mean (x̄) and range correlations from Leave-one-out (LOO) cross-validation are in italics. Variables are fully described in Materials and Methods, models and associated hypotheses are described in Table 1. LOO R code: https://figshare.com/articles/Supplement_2_R_code_used_for_wolf_analysis_/3550839

	Denning (n = 6)			Rendezvous (n = 6)			Nomadic (n = 4)		
Variables	β	±95%CI	+|-	β	±95%CI	+|-	β	±95%CI	+|-
E.Seral	**-1.4**	**0.09**	**2|1**	**0.6**	**0.1**	**3|0**	1.0	0.2	2|0
fLee	0.1	0.3	1|0	**-0.5**	**0.06**	**0|3**	**-0.9**	**0.07**	**0|3**
fMixed	0.1	0.9	1|1	-0.04	2.0	0|0	**1.2**	**0.1**	**3|0**
CTI	**0.2**	**0.03**	**4|0**	0.1	0.04	3|0	**0.2**	**0.04**	**3|0**
Elev	**-0.3**	**0.2**	**0|2**	**-0.4**	**0.08**	**0|4**	-0.2	0.4	0|1
Slope	**-0.1**	**0.2**	**1|3**	**-0.2**	**0.08**	**0|3**	**-0.3**	**0.1**	**0|3**
Dist	**0.2**	**0.07**	**2|0**	-0.3	0.04	0|4	0.2	0.1	1|0
A1k	**-0.3**	**0.2**	**2|0**	**-0.2**	**0.06**	**0|2**	-0.08	0.4	0|0
DST_W1M	**-0.2**	**0.1**	**1|3**	**-0.3**	**0.04**	**1|2**	**-0.3**	**0.08**	**2|0**
TPI	-0.02	0.6	1|2	-0.04	0.4	0|2	**0.3**	**0.1**	**2|0**
Lin70	0.06	0.3	1|1	**0.2**	**0.02**	**2|1**	0.08	0.3	0|0
DST_W20k	**-0.3**	**0.07**	**1|3**	**-0.1**	**0.1**	**1|3**	**-0.6**	**0.08**	**0|3**
fFlat	**-1.6**	**0.2**	**2|2**	**-4.3**	**0.03**	**0|1**	**-7.6**	**0.04**	**0|2**
WAM	-	-	-	0.1	0.1	2|0	-0.02	1.8	0|1
WAM:Dist	-	-	-	**0.2**	**0.09**	**3|0**	-0.2	0.1	0|0
E.Seral:Dist	-	-	-	-	-	-	0.5	0.4	2|0
E.Seral:WAM	-	-	-	-	-	-	-0.1	1.3	0|0
E.Seral:WAM:Dist	-	-	-	-	-	-	**0.7**	**0.5**	**1|0**
x̄ correlation (range)	*0.4 (0.2 - 0.9)*			*0.5 (0.2 - 0.8)*			*0.4 (0.1 – 0.6)*		

Appendix S6

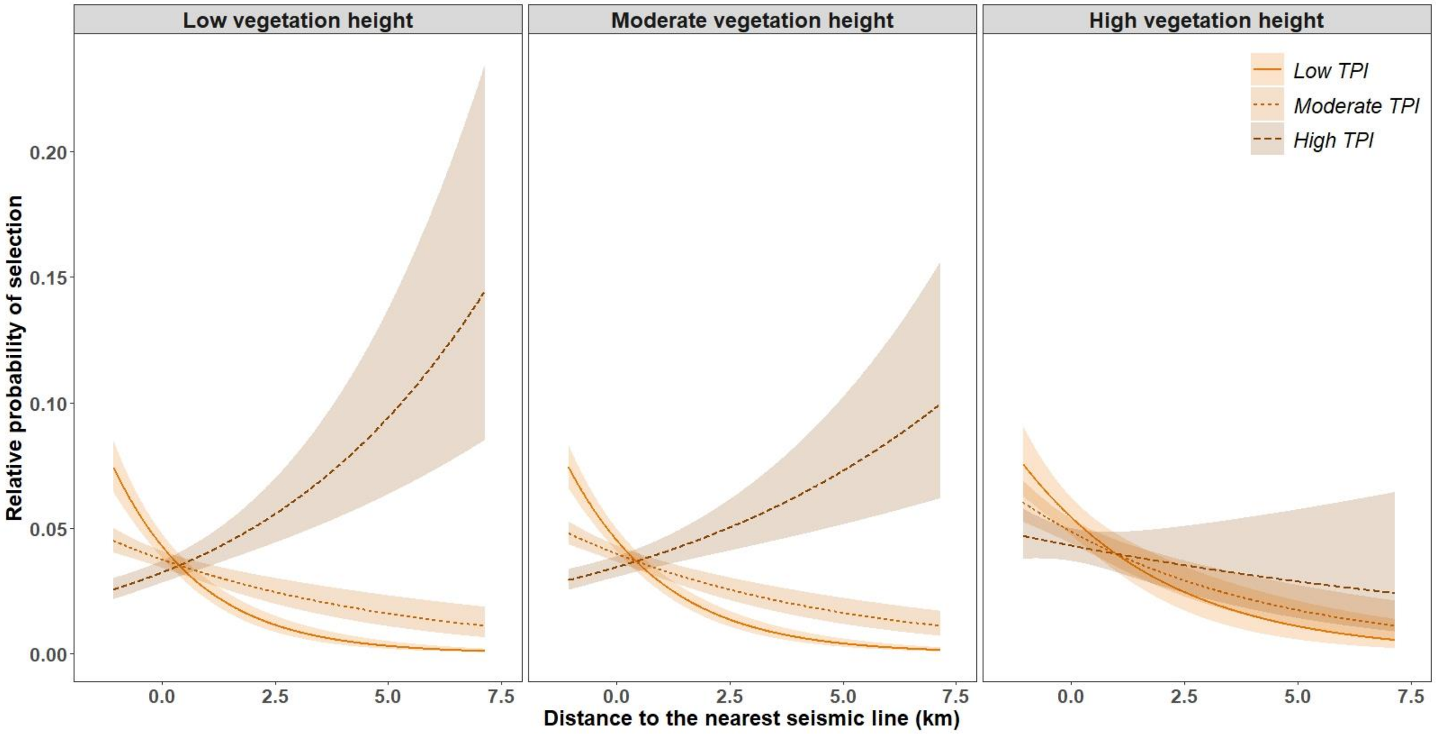

Fig. S1. Relative probability of selection from the most-supported model for wolves resting or feeding in the less-industrialized foothills landscape during the nomadic season in west-central Alberta, Canada between 2003-2009. Shaded areas are 95% prediction intervals. Each predictor variable is plotted within its observed range while other variables are held at their mean. *VegHT* and *TPI* are binned into low, moderate, and high categories based on quantiles for visual interpretation, models were built with continuous variables.

Appendix S7

Table S10. Population-level parameter estimates (β) and 95% confidence intervals (±95%CI) for influential interactions of additional models with weight of evidence (ω_i) ≥ 0.1 for each landscape-season-behaviour (less-industrialized vs. more-industrialized landscape; resting – feeding vs. travelling behaviour; denning vs. rendezvous vs. nomadic seasons). Best selected models for each landscape-season-behaviour investigating wolf selection near regenerating seismic lines in west-central Alberta, Canada between 2003 – 2009 are shown in Appendix S4. Models with no influential interactions are not shown. The number of individuals with significant positive (+) and negative (-) coefficients (95%CI not overlapping zero) are also shown. Variables are fully described in Materials and Methods, models and associated hypotheses are described in Table 1.

<table>
<tr><th></th><th></th><th colspan="3">Denning</th><th colspan="3">Rendezvous</th><th colspan="3">Nomadic</th></tr>
<tr><th>Models</th><th>Interactions</th><th>β</th><th>±95%CI</th><th>+|-</th><th>β</th><th>±95%CI</th><th>+|-</th><th>β</th><th>±95%CI</th><th>+|-</th></tr>
<tr><td colspan="11">More industrialized boreal landscape, resting - feeding</td></tr>
<tr><td>M6</td><td>Elev:VegHT:Dist</td><td>-</td><td>-</td><td>-</td><td>-</td><td>-</td><td>-</td><td>0.2</td><td>0.04</td><td>2|1</td></tr>
<tr><td></td><td>VegHT:Dist</td><td>-</td><td>-</td><td>-</td><td>-</td><td>-</td><td>-</td><td>0.4</td><td>0.04</td><td>5|0</td></tr>
<tr><td></td><td>Elev:VegHT</td><td>-</td><td>-</td><td>-</td><td>-</td><td>-</td><td>-</td><td>0.2</td><td>0.05</td><td>2|3</td></tr>
<tr><td></td><td>Elev:Dist</td><td>-</td><td>-</td><td>-</td><td>-</td><td>-</td><td>-</td><td>-0.1</td><td>0.09</td><td>0|3</td></tr>
<tr><td colspan="11">More industrialized boreal landscape, travelling</td></tr>
<tr><td>M6</td><td>Elev:VegHT:Dist</td><td>0.3</td><td>0.06</td><td>3|0</td><td>-</td><td>-</td><td>-</td><td>-</td><td>-</td><td>-</td></tr>
<tr><td></td><td>Elev:VegHT</td><td>0.2</td><td>0.2</td><td>1|1</td><td>-</td><td>-</td><td>-</td><td>-</td><td>-</td><td>-</td></tr>
<tr><td></td><td>Elev:Dist</td><td>-0.3</td><td>0.1</td><td>0|2</td><td>-</td><td>-</td><td>-</td><td>-</td><td>-</td><td>-</td></tr>
<tr><td>M4</td><td>A70:Dist</td><td>-</td><td>-</td><td>-</td><td>-</td><td>-</td><td>-</td><td>-0.3</td><td>0.2</td><td>0|1</td></tr>
<tr><td colspan="11">Less industrialized foothills landscape, travelling</td></tr>
<tr><td>M6</td><td>Elev:VegHT:Dist</td><td></td><td></td><td></td><td>0.1</td><td>0.08</td><td>1|1</td><td>-</td><td>-</td><td>-</td></tr>
<tr><td></td><td>Elev:VegHT</td><td></td><td></td><td></td><td>-0.4</td><td>0.08</td><td>0|2</td><td></td><td></td><td></td></tr>
<tr><td>M10</td><td></td><td></td><td></td><td></td><td></td><td></td><td></td><td></td><td></td><td></td></tr>
<tr><td></td><td>WAM:Dist</td><td></td><td></td><td></td><td></td><td></td><td></td><td>-0.1</td><td>0.03</td><td>0|2</td></tr>
</table>
